# Frank’s Sign Coexisting With a Giant Right Atrial Myxoma: A Case Report

**DOI:** 10.7759/cureus.86984

**Published:** 2025-06-29

**Authors:** Kotaro Mukasa, Shinichiro Abe, Yasunori Yakita, Soichi Asano

**Affiliations:** 1 Department of Cardiovascular Surgery, Chiba Cardiovascular Center, Ichihara, JPN

**Keywords:** cytokines, diagonal earlobe crease, frank’s sign, heart neoplasms, right atrial myxoma

## Abstract

Frank’s sign, a diagonal earlobe crease linked to atherosclerotic disease, remains poorly understood, and no association with cardiac tumors has been reported. We describe the case of a 68-year-old man who presented with exertional dyspnea and an elevated N-terminal pro-B-type natriuretic peptide level of 2,251 pg/mL. Transthoracic echocardiography identified a 75 mm × 50 mm pedunculated mass in the right atrium that prolapsed toward the tricuspid valve. Frank’s sign was present bilaterally despite the absence of coronary or peripheral arterial disease. Contrast-enhanced CT confirmed attachment of a gelatinous mass to the atrial free wall, and coronary CT angiography demonstrated no stenosis. The tumor was excised via median sternotomy under cardiopulmonary bypass established with aorto-superior vena cava cannulation, supplemented by a femoral venous cannula to facilitate inferior vena cava drainage. Histology showed spindle cells in a myxoid matrix without atypia, consistent with benign myxoma. To our knowledge, this is the first report describing the coexistence of Frank’s sign and cardiac myxoma. We speculate that tumor-derived cytokines such as interleukin-6 and vascular endothelial growth factor might impair earlobe microcirculation; however, coincidental coexistence cannot be excluded.

## Introduction

Frank’s sign is a diagonal crease on the earlobe running at approximately a 45-degree angle from the tragus to the lower margin of the lobule, and is reported to be associated not only with coronary artery disease (CAD) but also with cerebrovascular and peripheral arterial diseases [1]. Proposed mechanisms include microvascular injury, chronic inflammation, connective-tissue degeneration, and premature aging, yet its pathogenesis remains incompletely understood.

Primary cardiac tumors are rare, with an autopsy prevalence of 0.001-0.03% [2]. Among them, myxomas comprise roughly half; they are usually benign yet clinically important because of their potential for obstruction, arrhythmia, and embolization. Most myxomas arise in the left atrium, whereas right-atrial tumors account for only 15-20% of cases [3].

The typical clinical presentation of a right atrial myxoma includes a triad of symptoms: (1) signs of right-sided heart failure, such as exertional dyspnea, peripheral edema, and hepatomegaly; (2) embolic events, including pulmonary embolism caused by tumor or thrombus fragments; and (3) constitutional symptoms, resembling systemic inflammation due to cytokine release [4]. The differential diagnosis for a right atrial mass includes thrombus, metastatic tumors, primary malignant tumors, lipoma, and infective endocarditis [5]. Therefore, accurate diagnosis requires multimodal imaging. Transthoracic and, when necessary, transesophageal echocardiography are first-line modalities for assessing tumor size, stalk, and mobility, while CT and cardiac MRI are useful for tissue characterization and surgical planning [6]. If left untreated, there is a risk of valve obstruction, malignant arrhythmia, massive pulmonary embolism, and sudden death; thus, prompt surgical excision is recommended upon diagnosis [7]. Recent reports indicate a perioperative mortality rate of less than 2%, with excellent long-term outcomes [8].

No previous reports have described an association between Frank’s sign and primary cardiac tumors, particularly myxomas. We present a case of a giant right-atrial myxoma in a patient who also exhibited a clear Frank’s sign.

## Case presentation

A 68-year-old man reported several weeks of exertional dyspnea. Laboratory tests at the referring clinic revealed a N-terminal pro-B-type natriuretic peptide level of 2,251 pg/mL, and transthoracic echocardiography demonstrated an intracavitary right atrial mass. Therefore, he was referred for surgery.

**Figure 1:**
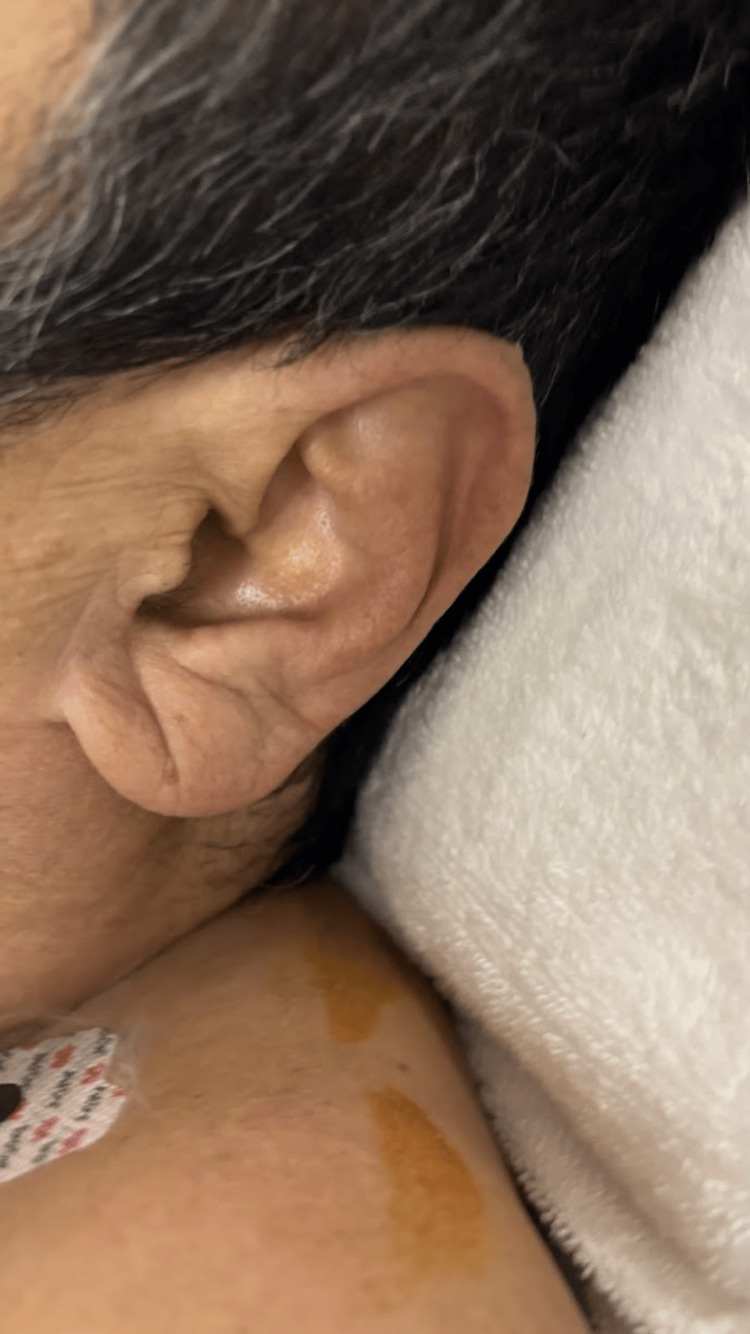
Photograph of Frank’s sign. Image showing Frank’s sign diagonally extending from the anterior to the posterior aspect of the patient’s left earlobe. Frank’s sign was present bilaterally in this patient.

On examination, Frank’s sign was evident in both earlobes (Figure 1). The body mass index was 20.0 kg/m^2^, indicating that the patient was not obese. The patient had no history or clinical evidence of CAD or peripheral vascular disease and had stopped smoking 38 years earlier. His medical history included catheter ablation for atrial fibrillation at another institution six years previously. Table 1 presents the laboratory data.

**Table 1:** Laboratory data on admission.

Items	Patient values	Reference ranges and units
Hemoglobin	12.3 g/dL	12.0–16.0 g/dL
White blood cell count	8.1 × 10⁹/L	4.0–10.0 × 10⁹/L
Platelet count	282 × 10⁹/L	150–400 × 10⁹/L
Serum creatinine	0.84 mg/dL	0.65–1.07 mg/dL
C-reactive protein	6.0 mg/dL	0.0–0.1 mg/dL
Low-density lipoprotein cholesterol	101 mg/dL	65–163 mg/dL
Triglyceride	61 mg/dL	40–234 mg/dL
N-terminal pro-B-type N-terminal-pro-B-type natriuretic peptide	2,251 pg/mL	0–125 pg/mL
Hemoglobin A1c	6.70%	4.9–6.0 %

**Figure 2:**
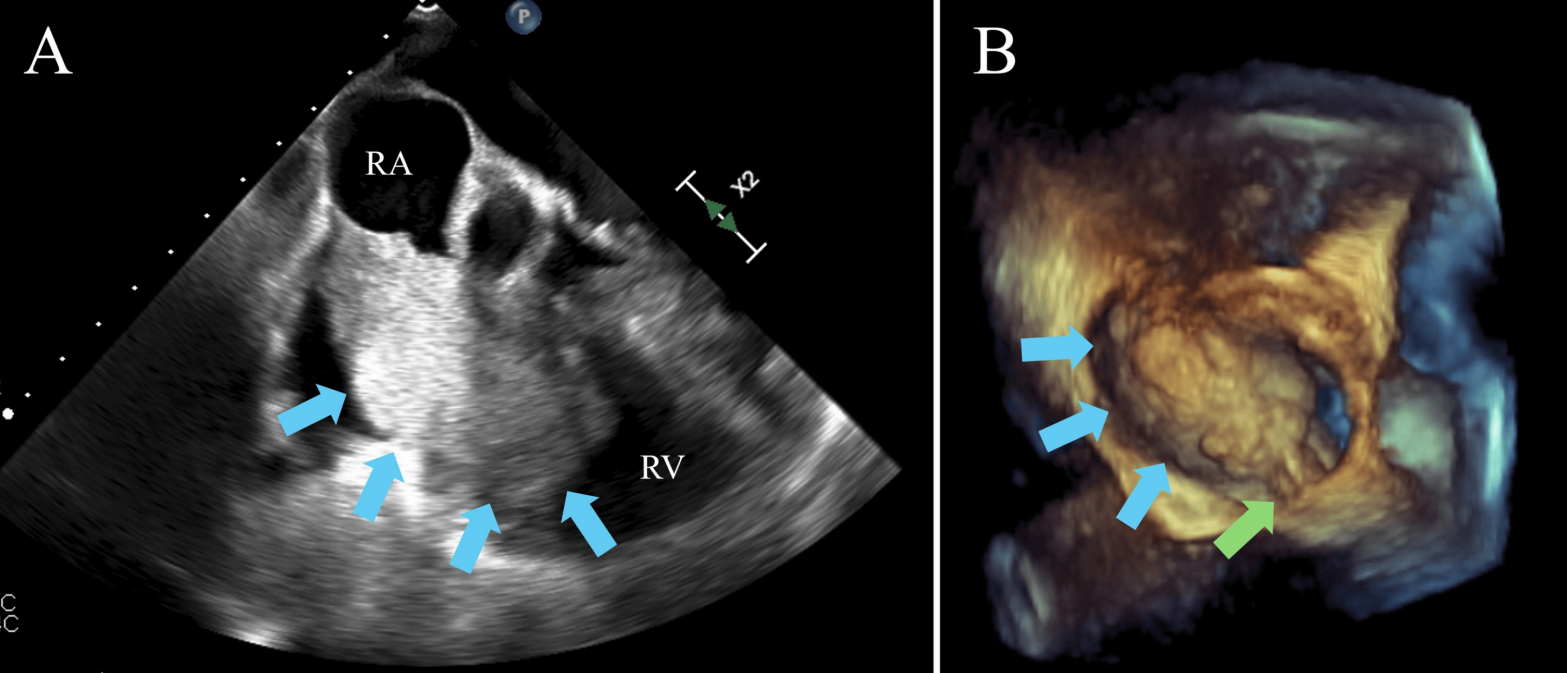
Transesophageal echocardiography images. (A) The tumor nearly prolapsing toward the tricuspid valve. (B) Three-dimensional reconstructed image showing a pedunculated tumor originating from within the right atrium. Blue arrow: tumor; green arrow: pedicle.

**Figure 3:**
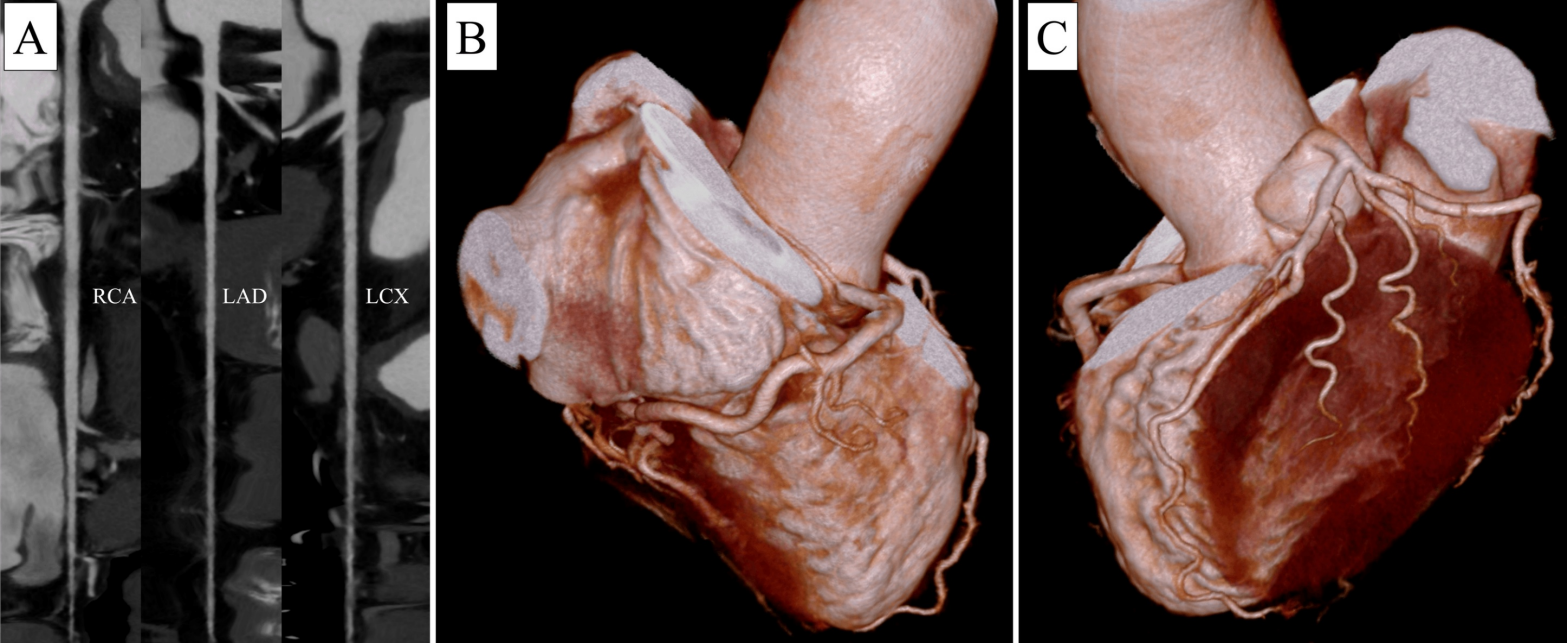
CT angiography image. CT angiography image showing no stenosis in the coronary arteries. (A) CT angiography showing the three main coronary arteries reconstructed from contrast-enhanced CT images. (B) Three-dimensional reconstructed image of the right coronary artery region. (C) Three-dimensional reconstructed image of the left coronary artery region.

**Figure 4:**
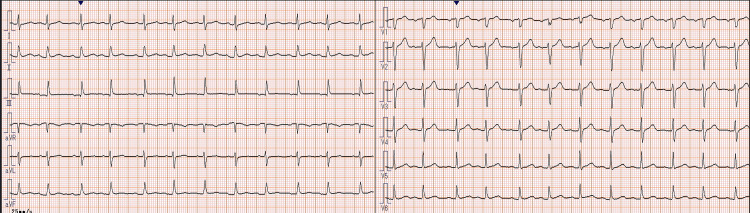
The 12-lead electrocardiogram on admission to our hospital. The 12-lead electrocardiogram on admission to our hospital showed no findings suggestive of ischemic heart disease, such as ST changes. The rhythm was sinus.

Transesophageal echocardiography confirmed a 75 mm × 50 mm pedunculated, highly mobile tumor originating from the right atrium and intermittently prolapsing toward the tricuspid valve (Figure 2). A patent foramen ovale with right-to-left shunting produced hemodynamics analogous to tricuspid stenosis with elevated right atrial pressure. Coronary CT angiography showed no significant stenosis (Figure 3). The ankle-brachial index was 1.14 on the right and 1.21 on the left. Hemoglobin A1c was 6.7 %, meeting the diagnostic threshold for diabetes mellitus, although the condition had not been recognized previously. Triglycerides were 61 mg/dL, and low-density lipoprotein-cholesterol was 98 mg/dL, showing no evidence of dyslipidemia. The patient also had no history of taking statins or other lipid-lowering medications. The electrocardiogram showed sinus rhythm, with no ST changes or other findings suggestive of ischemic heart disease (Figure 4). Owing to the risk of tricuspid valve obstruction, urgent surgical excision was scheduled.

**Figure 5:**
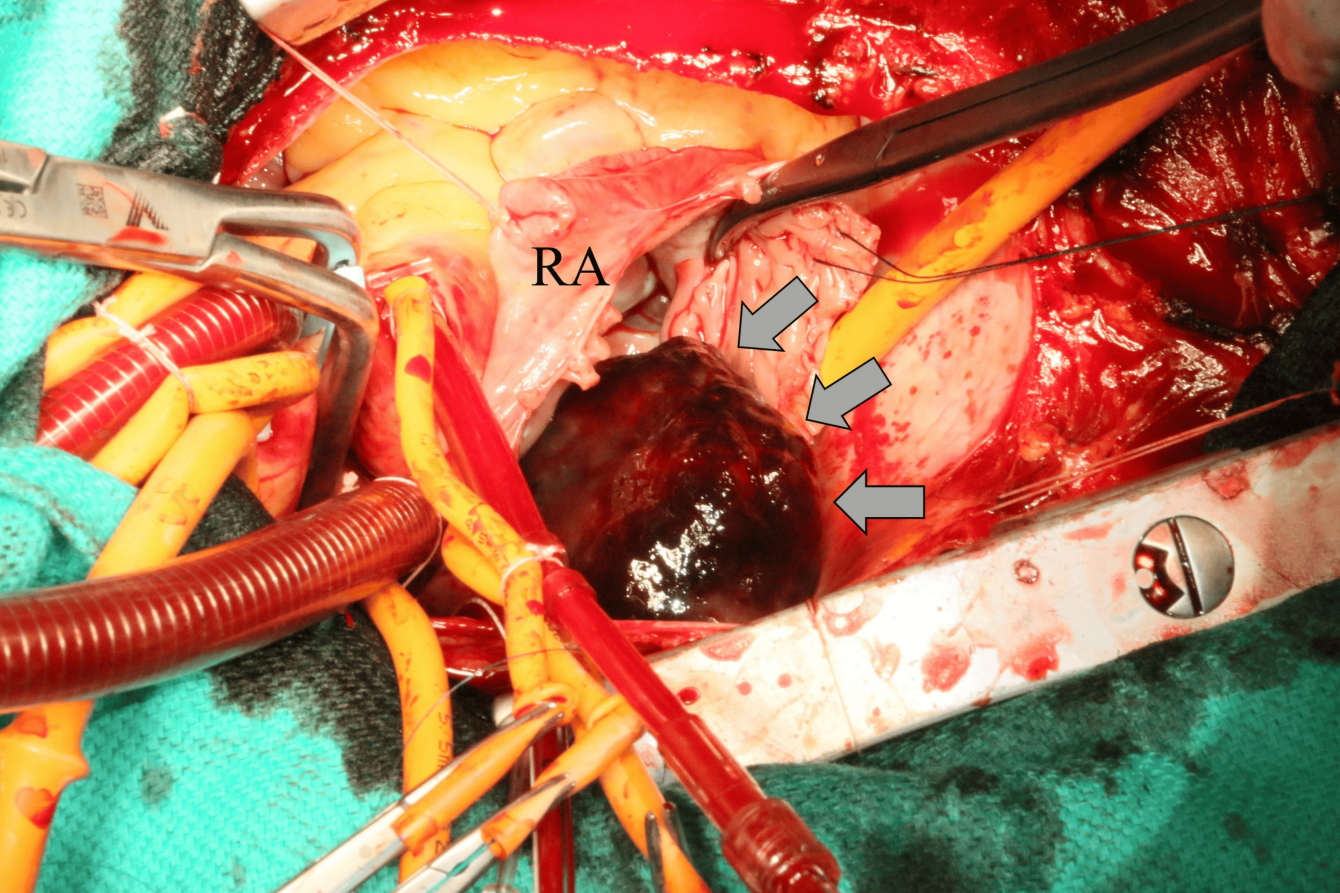
Intraoperative image of the right atrium tumor. Intraoperative image of the right atrium showing the tumor (grey arrow), viewed from the surgeon’s perspective. RA: right atrium

Under general anesthesia, a median sternotomy was performed. Cardiopulmonary bypass was established with aortic cannulation of the ascending aorta and venous drainage from the superior vena cava. An additional cannula inserted percutaneously into the right femoral vein was advanced to just below the atriocaval junction to avoid tumor fragmentation. After aortic cross-clamping, antegrade cardioplegia achieved cardioplegic arrest. Right atriotomy revealed a dark, gelatinous mass attached by a stalk to the free wall (Figure 5). The tumor and its pedicle were completely excised, and the patent foramen ovale was closed with polypropylene suture. The tricuspid valve appeared normal on visual inspection. A saline insufflation test was performed, and no significant regurgitation was observed.

The postoperative course was uneventful. Echocardiography on postoperative day five showed no residual mass, and the patient was discharged on day 10. Histopathological examination demonstrated spindle-shaped cells embedded in a myxoid stroma without nuclear atypia or mitotic figures, confirming benign myxoma.

## Discussion

Since Frank’s initial description in 1973, Frank’s sign has been regarded as a clinical marker of occult atherosclerotic disease, particularly in patients younger than 65 years of age [1,9]. Reported sensitivities and specificities, however, vary widely, and the sign rarely alters CAD management on its own [10]. The earlobe is supplied by end-arteries and lacks collateral circulation, making it susceptible to microvascular injury, chronic hypoxia, and connective tissue degeneration [11]. Histopathological studies have shown myoelastofibrosis in the arteries at the base of the crease and Wallerian-like degeneration in peripheral nerves [12]. There are also reports suggesting a mechanical etiology related to visceral facial fat [13]. However, the precise mechanism remains unclear.

In the present case, obvious atherosclerotic disease was absent: coronary CT showed neither calcification nor stenosis, and ankle-brachial index values were normal. Although HbA1c met the diagnostic threshold for diabetes mellitus, the patient had not been diagnosed previously, and his smoking exposure was remote. A literature search revealed no previous descriptions of coexistent Frank’s sign and cardiac myxoma.

If a true association exists, tumor-derived cytokines such as interleukin-6 and vascular endothelial growth factor could conceivably damage the earlobe microcirculation by promoting systemic inflammation, angiogenesis, and endothelial dysfunction [14].

Frank’s sign has been linked to several non-atherosclerotic conditions, including atrial fibrillation and acute aortic dissection [15,16], both characterized by inflammation, oxidative stress, or vasa vasorum ischemia [17]. These observations suggest that Frank’s sign may reflect chronic cardiovascular stress rather than atherosclerosis alone. Clinicians who detect Frank’s sign should, therefore, remain alert not only to CAD but also to other potential sources of sustained cardiovascular insult, including cardiac tumors.

This report describes a single case, and no causal relationship between Frank’s sign and cardiac myxoma can be established. Given the relatively high prevalence of Frank’s sign in older adults and the rarity of cardiac myxomas, their coexistence in this case may be coincidental. Further studies are needed to clarify whether any pathophysiological or epidemiological association exists.

## Conclusions

This report describes a case of Frank’s sign coexisting with a cardiac myxoma, notably in a patient lacking evident atherosclerotic disease. To our knowledge, such a detailed coexistence has not been previously documented. This unusual association suggests that the pathogenesis of Frank’s sign may extend beyond atherosclerosis, with one speculative mechanism being tumor-derived cytokine-mediated microvascular injury in the earlobe. This case suggests that Frank’s sign may represent a broader indicator of chronic cardiovascular stress than previously thought, and that clinicians should consider not only ischemic heart disease but also cardiac tumors in the differential diagnosis. Further investigation is essential to fully elucidate the underlying pathophysiology and diverse clinical significance of Frank’s sign.
